# Probing SH2-domains using Inhibitor Affinity Purification (IAP)

**DOI:** 10.1186/1477-5956-12-41

**Published:** 2014-07-16

**Authors:** Michael Höfener, Stephanie Heinzlmeir, Bernhard Kuster, Norbert Sewald

**Affiliations:** 1 Organic and Bioorganic Chemistry, Department of Chemistry, Bielefeld University, Universitätsstraße 25, 33615 Bielefeld, Germany; 2 Chair for Proteomics and Bioanalytics, Center of Life and Food Sciences Weihenstephan, Technische Universität München, Freising, Germany; 3 German Cancer Consortium (DKTK), Munich, Germany; 4 German Cancer Research Center (DKFZ), Heidelberg, Germany; 5 Center for Integrated Protein Science Munich, Freising, Germany

**Keywords:** Chemical proteomics, Inhibitor affinity purification, Mass spectrometry, PI3 kinase, SH2 domain

## Abstract

**Background:**

Many human diseases are correlated with the dysregulation of signal transduction processes. One of the most important protein interaction domains in the context of signal transduction is the Src homology 2 (SH2) domain that binds phosphotyrosine residues. Hence, appropriate methods for the investigation of SH2 proteins are indispensable in diagnostics and medicinal chemistry. Therefore, an affinity resin for the enrichment of all SH2 proteins in one experiment would be desirable. However, current methods are unable to address all SH2 proteins simultaneously with a single compound or a small array of compounds.

**Results:**

In order to overcome these limitations for the investigation of this particular protein family in future experiments, a dipeptide-derived probe has been designed, synthesized and evaluated. This probe successfully enriched 22 SH2 proteins from mixed cell lysates which contained 50 SH2 proteins. Further characterization of the SH2 binding properties of the probe using depletion and competition experiments indicated its ability to enrich complexes consisting of SH2 domain bearing regulatory PI3K subunits and catalytic phosphoinositide 3-kinase (PI3K) subunits that have no SH2 domain.

**Conclusion:**

The results make this probe a promising starting point for the development of a mixed affinity resin with complete SH2 protein coverage. Moreover, the additional findings render it a valuable tool for the evaluation of PI3K complex interrupting inhibitors.

## Background

Many human diseases are caused by imbalanced regulation of crucial cellular processes. Cell metabolism is regulated via a multitude of complex signal transduction cascades. Signals derived from extracellular stimuli are forwarded by phosphorylation events, which need to be strictly controlled. Dysregulation of these signaling processes can cause many human diseases such as cancer [1]. Different protein kinases are key regulators of the corresponding regulation cascades and, therefore, one of the most prominent research targets in drug development [2,3]. In the initial activation events many protein complexes are formed in order to initiate phosphorylation cascades that further activate transcription factors. Hence, the investigation of these complexes is of major importance and still remains challenging since the participating proteins are usually expressed at low levels [4,5]. There are many examples in the literature of kinase inhibitors that were applied in clinical studies or are available as approved drugs [6]. These kinase inhibitors usually address the ATP binding domain in the active (type 1 inhibitors) or inactive (type 2 inhibitors) conformation of the enzyme. The prohibition of protein interactions required for activation of signal transduction kinases is an alternative strategy for the direct inhibition. The SH2 (Src homology 2) domain that binds to phosphorylated tyrosine (pY) residues is one of the most important domains responsible for protein interaction. 120 SH2 domains are present in 110 distinct human proteins according to the UniProt database and Liu et al. [7-9]. SH2 domain mediated PI3K heterodimer formation is a prominent example of such a protein interaction. PI3 kinases participate in signal transduction by generating second messengers that further activate multiple effector pathways, including Akt-, NF-κB- and Jnk-signaling. PI3 kinases are correlated with various diseases such as cancer [10]. Taking this aspect into account, it is not surprising that addressing the SH2 binding domain is an important strategy for therapeutic purposes [11]. A mixed kinase affinity resin for the evaluation of ATP competitive kinase inhibitors, called Kinobeads [12] is available and a comparable affinity-based approach for the evaluation of SH2 addressing inhibitors would be desirable. Kinobeads consist of different affinity probes immobilized on a solid phase and have been proven to capture a major part of the human kinome by addressing the ATP binding pocket. They are used to investigate the kinase target spectrum and for the determination of binding affinities in competition experiments with ATP competitive drugs [12]. A mixed SH2 domain affinity resin that addresses the majority of SH2 containing proteins would be desirable to adopt this methodology to SH2 proteins. Methods based on a variety of bait peptides (e.g. pY-peptide chips) have already been developed for the investigation of these protein-protein interactions [13,14]. However, coverage of the full range of SH2-proteins turned out to be problematic. 70 SH2 proteins could be addressed by utilizing a mixture of 6200 different immobilized 13-membered pY-peptides [14]. One disadvantage of this chip-based method is the requirement of SH2-GST fusion proteins for the analysis of the profiling experiments via anti-GST fluorescent antibodies. An inhibitor affinity purification (IAP) based approach was established for the investigation of the EGFR interactome that used at least 24 different immobilized pY-peptides for probing SH2 proteins [15,16]. The IAP approach with the highest amount of captured SH2 proteins used 57 13-membered pY-peptides immobilized on magnetic beads that captured 45 SH2 proteins [14]. However, the number of compounds immobilized in the IAP experiment inversely correlates with the effective concentration of each immobilized substance [17]. As a result low abundant SH2 protein members will not be enriched sufficiently. Hence, all described efforts to develop an IAP approach that covers all 110 human SH2 proteins were not successful. In this study a small pY containing dipeptide that only addresses the conserved pY-core motif addressing the SH2 domain was used as a probe for the IAP experiment to potentially overcome these limitations in the future.

## Results/discussion

### Design and synthesis

**Figure 1:**
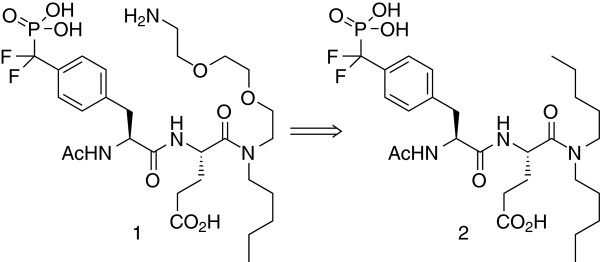
**Probe design.** Probe **1** is based on the peptide inhibitor **2**[18]. It can be immobilized on a solid support via the primary amine function of its linker moiety.

**Figure 2:**
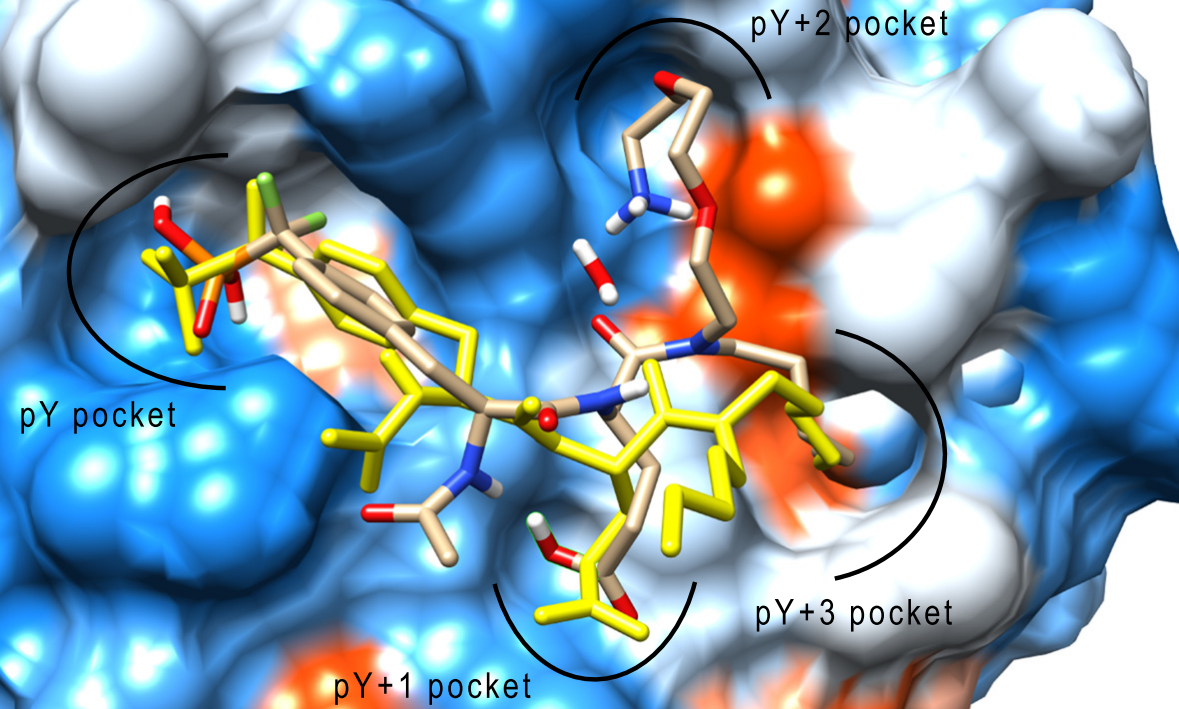
**Target binding properties of probe 1.** X-ray crystal structure of the pYE SH2 ligand **2**[18] (yellow) in complex with the c-Src-SH2 domain (PDB: 1a08). Probe **1** was docked to that protein structure and the docking pose incorporated into that complex (atom specific coloring).

For the IAP approach a linkable probe that carries a SH2 domain affinity group and allows for the immobilization on a solid support is required (Figure 1) [19]. SH2 domains recognize pY-peptides of at least three to eight residues and consist of two major binding pockets, the highly conserved pY pocket and the hydrophobic pY+3 pocket [20]. Both regions were shown to be essential for SH2 ligand binding and are, therefore, highly conserved [21]. The two point interaction mode induced by the conserved pY and pY+3 pocket is also known as “two-pronged plug two-holded socket” binding model [11]. In addition to the conserved binding regions further binding pockets are mainly responsible for the specificity of the ligand recognition. Hence, for the development of specific ligands such additional pockets are often addressed e.g. pY-2 to pY+3 (VIpYFVP) or pY-1 to pY+5 (GpYLPQTV) [22]. In addition, cyclic peptidomimetics that are less flexible have been developed for selective inhibition of distinct SH2 proteins [23]. In order to create a SH2 protein probe that is potent towards SH2 proteins but not selective for a specific protein a small peptide that only addresses pY to pY+3 seems appropriate for the inhibitor moiety. All natural SH2 ligands contain a pY residue and it was shown that the omission of the phosphate group decreased the binding affinity towards SH2 domains significantly. In general, a pY residue is essential for a potent probe and, therefore, has to be incorporated into the probe structure. Glutamic acid in pY+1 position is known to have a flexible binding pattern [21] and, as a result, seems to be a good residue for a mixed SH2-protein affinity resin. N-Acetylated phosphotyrosine residues are present in many SH2 inhibitors and, therefore, may be considered as potential moiety for the probe structure. The SH2 domain inhibitor **2** that was previously applied for a thermodynamic and structural study on SH2 domains [18] was chosen as starting point for the probe development, since it consists of several key moieties for a potent SH2 domain probe with broad SH2 protein affinity. Additionally, X-ray crystal structures of SH2 proteins co-crystallized with inhibitor **2** are available to determine potential linker modification sites. Another key feature of inhibitor **2** is its non-hydrolysable phosphotyrosine building block that was incorporated into the molecule without significant loss of biological activity. One of the inhibitor’s pentyl residues was chosen for linker modification, since it points outside the binding site. Molecular docking analysis was used to confirm similar target binding properties of probe **1** compared to inhibitor **2**[18]. The binding mode of the modified pYE SH2 ligand **1** (probe **1**) is in accordance with the original inhibitor **2** (Figure 2) [24]. Due to the chosen linker modification the pY+2 is rationally not addressed by the probe, but the attached alkyl chain addresses the pY+3 binding pocket. For the alkyl chain that addresses the pY+3 pocket, the small flexible n-pentyl chain as it can be found in inhibitor **2**, was kept in the probe molecule in order to cover a broad spectrum of SH2 proteins [25]. However, the results of the docking experiments revealed that the pY+2 pocket may be occupied by the oligoethyleneglycol linker (Figure 2). These findings indicate that probe **1** is a promising candidate for the IAP approach. As shown in Figure 2, the phosphonic acid is mainly stabilized by electrostatic interactions with the side chains of R158, R178, S180, T182 and T183 and the amide protons of R158, E181, T182 and T183 (amino acid positions adopted from PDB: 1a08). The aryl moiety of the pY residue is stabilized via cation-π-interactions with R158 and R178. The carbonyl groups of the N-acetyl group, the pY backbone and the glutamic acid side chain interact with H204. In addition, the carbonyl group of the glutamic acid side chain is also stabilized by electrostatic interactions with Y205. The pentyl residue binds to a hydrophobic pocket formed by Y205, I217, T218 and L240.

According to the synthesis described by Vu et al. [5], probe **1** can easily be obtained using manual solid phase peptide synthesis on a 2-chlorotrityl resin. The required F2pmp building block was directly synthesized by copper mediated cross coupling of an organozinc compound in analogy to published procedures [26-31].

### SH2 enrichment

**Figure 3:**
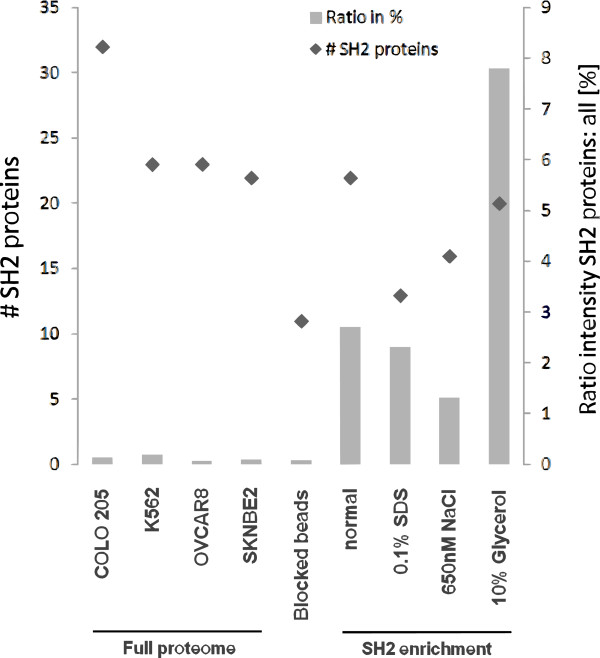
**SH2 protein enrichment.** The MS-intensity ratio of SH2 proteins in relation to all identified proteins (right y axis) and the number of identified SH2 proteins (left y axis). Displayed is full proteome data from Colo205, K562, Ovcar8 and SKNBE2 cells (without probe **1** enrichment), pull-down data applying blocked beads and pull-down data applying probe **1** to mixed cell lysates of Colo205, K562, Ovcar8 and SKNBE2 cells.

The probe was immobilized on sepharose beads and pull-down experiments were performed using mixed cell lysates (Colo205, K562, Ovcar8 and SKNBE2). Since the utilized cell lines feature different protein profiles, a mix of these lysates offers a versatile pool of proteins for pull-down experiments. This cell lysate mix was used for protein kinase enrichment before and performed well, which led to the conclusion, that it might also contain a high variety of other signaling-related proteins such as SH2 proteins. Therefore, this mixed cell lysate was chosen for the first pull-down experiments (Additional file 1). Captured proteins were eluted from the beads, digested with trypsin and identified using liquid chromatography tandem mass spectrometry (LC-MS/MS). This analysis gave 1557 proteins in total, including 22 SH2-proteins. SH2 proteins have been enriched with an intensity ratio of 2.7%, using summed intensities for SH2 proteins compared to the summed intensities for all other identified proteins from the MS experiment. In order to minimize unspecific binding the pull-down experiment was optimized (Additional file 2) using 10% glycerol in the washing step (Figure 3). The corresponding results included 1168 proteins in total, of which 20 were SH2-proteins with an intensity ratio of 7.8%. In the full proteome data of the single cell lines (without prior enrichment with probe **1**) the intensity ratio of SH2 proteins varies from 0.06% to 0.18%. This shows that the enrichment with probe **1** under optimized conditions with the mixed cell lysate increases the SH2 protein abundance by a factor of 43 to 87 (compare Figure 3). This clearly shows that SH2-proteins have successfully been enriched by probe **1**. Since the mixed cell lysate covers only 50 out of the 110 known SH2 domain containing proteins (Additional file 3: Figure S2) [9] and low abundant proteins get diluted by mixing, it is likely that the comparably small number of identified and enriched SH2 proteins is an issue of the chosen cell lines, rather than an issue of the probe. Taking additionally into account that SH2 proteins are generally less abundant (Additional file 3: Figure S1) underlines the value of probe **1** for SH2 protein enrichment. According to the data received by proteome analysis of the NCI-60 cell line panel [28] the cell line Colo205 features high SH2 protein content (Additional file 3: Figure S1). Therefore, it was chosen for further experiments. In order to confirm specific SH2 domain binding, pull-down experiments were performed with lysates derived from Colo205 cells treated with the phosphatase inhibitor pervanadate or water as the control (Additional file 4). The measured differences were quantified with intensity based label free quantification using the MaxQuant software. In the presence of pervanadate phosphorylated tyrosine residues should not be removed by phosphatases. Thus, SH2 domains are likely to be occupied by the excess of naturally occurring pY peptides and proteins competing with the immobilized bait. Hence, the amount of enriched SH2 proteins is decreased compared to the control experiment.

**Figure 4:**
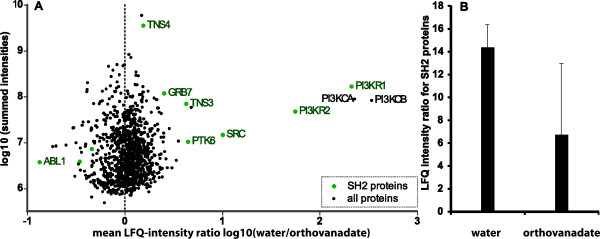
**Pull-down experiments applying probe 1 to cell lysates derived from Colo205 cells treated with pervanadate or water as the control (triplicates). A**: Volcano plot of the measured differences using intensity based label-free quantification and **B**: SH2 protein enrichment indicated by the SH2-protein LFQ intensity ratio from the MS experiments, displayed are medians of the intensity ratios of the pervanadate and water treated samples from the triplicate analysis. The error bars display the standard deviation between the replicates. The comparably high standard deviation in the pervanadate treated samples mainly originates from one protein in one of the replicates (for detailed data refer to supplementary information).

Although the Colo205 cell line seemed promising for further experiments, only 8 SH2 proteins out of 23 present in the Colo205 proteome [28] were successfully quantified in three replicates. However, PI3K, SRC and ABL1 kinase that contain structurally very similar SH2 domains [29] have been reproducibly enriched in all data sets. These findings indicate that the probe molecule could be optimized for SH2 proteins with structurally similar SH2 domains by varying the alkyl based pY+3 binding moiety [29]. According to the “two-pronged plug and socket” mechanism these results additionally imply that the probe structure could be varied for proteins containing other SH2 families by incorporating a different moiety at this position. The triplicate analysis of the intensity ratios of the SH2 proteins shows that the amount of enriched SH2 proteins is significantly lower (7%) for the pervanadate treated samples compared to 14% in the control experiment without pervanadate (compare Figure 4B). All identified SH2 proteins show a distinct difference between the pervanadate treated and the untreated conditions. This implies that the captured SH2 proteins bind specifically to the probe as expected. Additionally, in terms of LFQ intensities SH2 protein enrichment was slightly increased when using Colo205 cells only (Figure 4B) compared to the IAP experiment with mixed cell lysate (Figure 3). Although probe **1** was able to specifically enrich SH2 proteins and furthermore identified 22 different SH2 proteins, more effective SH2 probes are still needed to obtain complete SH2 protein coverage.

The PI3 kinases are targets of current research [10]. They generate lipid second messengers, act as membrane docking sites for various downstream effector proteins and consist of heterodimeric complexes of regulatory, SH2 domain containing subunits and catalytic subunits lacking SH2 domains. The catalytic domains p110α (PI3KCA), p110β (PI3KCB) and p110δ dimerize each with any of the regulatory subunits p85α (PI3KR1), p85β (PI3KR2), p55γ, p55α or p50α [32]. The regulatory subunits stabilize the thermally labile catalytic subunits and inhibit their catalytic activity. Upon cellular stimulation, these heterodimers are recruited to membrane proximal pY proteins and the catalytic subunits thereby activated. The catalytic subunits PI3KCA and PI3KCB as well as the corresponding regulatory subunits (three SH2 domains: [30] C-terminal, internal and N-terminal) PI3KR1 and PI3KR2 have been enriched by probe **1** under pervanadate-free conditions. The PI3K subunits show significantly high differences between the controls and the pervanadate depleted samples. These results imply that the regulatory subunits are captured specifically by an interaction of one of their three SH2 domains with probe **1**. Although the catalytic subunits do not have any SH2 domains, they bind to the probe and show the same behavior in the pervanadate experiments. This finding shows that the catalytic subunits are captured as complexes with the regulatory subunits also under pervanadate untreated conditions. Interestingly, the involved pY residues of the catalytic PI3K subunits seem to be relatively stable towards phosphatases, since the corresponding PI3KCA/PI3KR1 heterodimer can be enriched in the IAP experiment without phosphatase inhibition (Figure 4A). It is very likely that the capturing proceeds via the N-terminal SH2 domain, because this domain is not essential for the heterodimer formation and is structurally very similar to the SH2 domains of SRC and ABL1 [29] that were both enriched, too. These results of the depletion experiments show, that probe **1** could be applied to evaluate SH2 domain addressing inhibitors of the participating regulatory subunits of PI3 kinase complexes.

Another interesting result was obtained for the kinase ABL1 that contains a SH2 domain and was highly enriched in the pervanadate experiment (Figure 4A). One possible explanation for this phenomenon could be that the SH2 domain is conformationally masked depending on the phosphorylation state of ABL1 and therefore not enriched under pervanadate conditions. A similar correlation was previously shown for Tyr70 and the SH3 domain of ABL1 [31]. However, further experiments are required to investigate that hypothesis.

**Figure 5:**
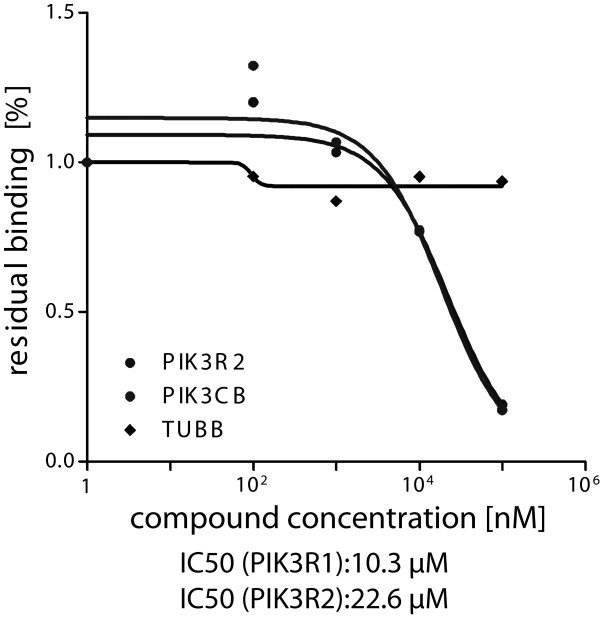
**Dose-response curves.** Pulldown experiments were conducted using probe **1** with Colo205 cell lysate and increasing concentrations of the free compound **1**.

A competitive binding experiment was performed to validate the results obtained under pervanadate-depletion conditions (Additional file 4). Dose response pull-down experiments were accomplished in Colo205 lysates with increasing concentrations of the free compound **1** that competes with the immobilized probe **1** for SH2 domain binding (Additional file 5). As depicted in Figure 5 probe **1** shows IC_50_ values in the low micromolar range (10-23 μM) for the captured regulatory PI3K subunits. These values are comparable with the data published for several SH2 domain addressing inhibitors (3-50 μM) [4,33,34]. Additionally, these findings underscore the results of the pervanadate depletion experiments that indicate that the regulatory PI3 kinase subunits specifically interact with probe **1** via an SH2 domain. The regulatory PI3K subunits PI3KR1 and PI3KR2 contain a C-terminal and an N-terminal SH2 domain. The real IC_50_ values are likely smaller than the measured values for PI3KR1 and PI3KR2, since it is impossible to distinguish whether one of the two domains or both simultaneously interact with equal affinity with probe **1**[35]. As expected all identified SH2 proteins show a dose dependent decrease in the competition experiment, whereas the other proteins such as tubulin beta chain (TBB) that is one of the most abundant proteins in the cell and in the pull-down results, do not show dose dependent decrease. These results render probe **1** a valuable tool for the investigation of PI3K subunit interactions and additionally underline the potency of probe **1** to bind SH2 domains.

## Conclusions

Probe **1** selectively enriches SH2 proteins and allows for the identification of 22 of the 50 different SH2 proteins abundant in the chosen lysate with only one substance. The previously published IAP approach with the highest amount of captured SH2 proteins used 57 13-membered pY-peptides immobilized on magnetic beads and captured 45 SH2 proteins [14]. Therefore, we strongly believe that our probe is already an improvement and should be considered as starting point for a full coverage SH2 protein resin. Such resin could be used for further SH2 inhibitor design or for the investigation of SH2 protein interactions. Additionally it could be applied to detect changes in the SH2 proteome of distinct tissues or cells. Since probe **1** captures two PI3 kinase complexes it can be applied for the investigation of the participating SH2 interactions and the evaluation of PI3K complex interrupting inhibitors. However, a more sufficient cell lysate for SH2 protein enrichment would be desirable.

## Methods

### Statistical analysis

A two-tailed student’s t-test assuming equal variance was performed with log transformed LFQ intensities to obtain p-values for each protein (p = 0.01 highly significant, p = 0.05 significant).

### Docking experiment

The docking calculations were performed using AutoDock Vina and AutoDock Tools [24]. All dockings were conducted as blinds using a search space that fits the whole protein structure. The figure was built using UCSF Chimera 1.7 [36].

### Sample preparation

Cells were cultivated in humidified air supplemented with 5% CO_2_ at 37°C using Roswell Park Memorial Institute 160 medium (RPMI1640) for the K562 (DSMZ, Deutsche Sammlung von Mikroorganismen und Zellkulturen GmbH, Braunschweig, Germany) and Colo205 (CLS, Cell Lines Service, Eppelheim, Germany) cells, Dulbecco’s modified Eagle’s medium (DMEM) for OVCAR8 (Anderson, Matthew L, Department of Pathology, Baylor College of Medicine, Houston, Texas) and SKNBE2 (DSMZ, Deutsche Sammlung von Mikroorganismen und Zellkulturen GmbH, Braunschweig, Germany) cells and Iscove’s modified Dulbecco’s medium (IMDM) for HeLa013 cells. All media were supplemented with 10% fetal bovine serum (FBS).

For pervanadate treatment, 100 mM sodium vanadate and 100 mM hydrogen peroxide solution were mixed in equal amounts resulting in a 50 mM pervanadate solution. The pervanadate solution was added to the cell culture medium to a final concentration of 100 μM and incubated for 25 min at 37°C.

Cells were washed with cold phosphate buffered saline (PBS) and harvested by lysis using 50 mM Tris/HCl pH 7.5, 5% Glycerol, 1.5 mM MgCl_2_, 150 mM NaCl, 0.8% NP-40, 1 mM dithiothreitol and 25 mM NaF with freshly added protease inhibitors and phosphatase inhibitors (5× phosphatase inhibitor cocktail 1, Sigma-Aldrich, Munich, Germany, 5× phosphatase inhibitor cocktail 2, Sigma-Aldrich, Munich, Germany, 1 mM Na_3_VO_4_ and 20 nM calyculin A, LC Laboratories, Woburn, MA, USA). Protein extracts were clarified by centrifugation for 20 min at 100,000 × g at 4°C and protein concentration was determined by the Bradford method.

### Compound coupling

Probe **1** (Additional file 3) was immobilized on sepharose beads through covalent linkage using its primary amino group [12]. 1 ml of NHS-activated sepharose (GE Healthcare, Freiburg, Germany) and the compound (2 μmol/mL beads) were equilibrated in DMSO. 15 μl of triethylamine was added to start the coupling reaction and the mixture was incubated on an end-over-end shaker for 16-20 h in the dark. Free NHS-groups on the beads were blocked by adding 50 μl aminoethanol and incubation on an end-over-end shaker for 16-20 h in the dark. Coupled beads were washed and stored in ethanol at 4°C in the dark. The coupling reaction was monitored by LC-MS.

### Affinity purification

SH2 probe pulldowns were performed as described previously [12]. Briefly, cell lysates were diluted with equal volumes of 1× compound pulldown (CP) buffer (50 mM Tris/HCl pH 7.5, 5% glycerol, 1.5 mM MgCl_2_, 150 mM NaCl, 25 mM NaF, 1 mM dithiothreitol and freshly added protease inhibitors and phosphatase inhibitors (5× phosphatase inhibitor cocktail1, Sigma-Aldrich, Munich, Germany, 5× phosphatase inhibitor cocktail 2, Sigma-Aldrich, Munich, Germany, 1 mM sodium pervanadate and 20 nM calyculin A, LC Laboratories, Woburn, MA, USA)). For the dose response experiment, lysates were pre-incubated with the free compound for 30 min at 4°C. Lysates were incubated with beads for 30 min at 4°C. Subsequently, the beads were washed with 1× CP buffers containing 0.4% and 0.2% NP40 and 10% glycerol. Bound proteins were eluted with 2× NuPAGE LDS Sample Buffer (Invitrogen, Darmstadt, Germany) and eluates were reduced and alkylated by 50 mM dithiothreitol and 55 mM chloroacetamide. Samples were then run into a 4–12% NuPAGE gel (Invitrogen, Darmstadt, Germany) for about 0.5 cm to concentrate the sample prior to in-gel tryptic digestion. In-gel trypsin digestion was performed according to standard procedures.

### LC-MS/MS measurements

Nanoflow LC-MS/MS was performed by coupling an Eksigent nanoLC-Ultra 1D + (Eksigent, Dublin, CA) to a LTQ-Orbitrap XL ETD (Thermo Scientific, Bremen, Germany). Peptides were delivered to a trap column (100 μm × 2 cm, packed in-house with Reprosil-Pur C_18_-AQ 5 μm resin, Dr. Maisch, Ammerbuch, Germany) at a flow rate of 5 μL/min in 100% solvent A (0.1% formic acid in HPLC grade water). After 10 min of loading and washing, peptides were transferred to an analytical column (75 μm × 40 cm, packed in-house with Reprosil-Pur C_18_-GOLD, 3 μm resin, Dr. Maisch, Ammerbuch, Germany) and separated using a 210 min gradient from 4% to 32% of solvent B (0.1% formic acid, 5% DMSO in acetonitrile; solvent A: 0.1% formic acid, 5% DMSO in water) at 300 nL/minute flow rate. The LTQ Orbitrap XL was operated in data dependent mode, automatically switching between MS and MS [2]. Full scan MS spectra were acquired in the Orbitrap at 60,000 (m/z 400) resolution after accumulation to a target value of 1,000,000. Tandem mass spectra were generated for up to eight peptide precursors in the linear ion trap by using collision-induced dissociation at a normalized collision energy of 35% after accumulation to a target value of 5,000 for max 100 ms.

### Peptide and protein identification and quantification

Raw MS data were processed by MaxQuant (v1.4.0.5) for peak detection and quantification [37] MS/MS spectra were searched against the UniProtKB human database (22.07.13, 88.354 sequences) using the Andromeda search engine [38] with the following search parameters: full tryptic specificity, re-quantification and match-between-runs option, up to two missed cleavage sites, carbamidomethylation of cystein residues was set as a fixed modification and N-terminal protein acetylation, methionine oxidation and phosphorylation as variable modifications. Mass spectra were re-calibrated within MaxQuant (first search 20 ppm precursor tolerance) and subsequently re-searched with a mass tolerance of 10 ppm. Fragment ion mass tolerance was set to 0.5 Da. Search results were filtered to a maximum false discovery rate (FDR) of 0.01 for proteins and peptides and a peptide length of at least 7 amino acids was required.

LFQ intensities of 0 (in control replicates) were replaced with the 1% percentile of the LFQ intensities of this respective measurement for data visualization.

## Abbreviations

ATP: Adenosine triphosphate; SRC: Proto-oncogene tyrosine-protein kinase SRC; SH2: SRC homology 2; AKT: Serine/threonine-protein kinase AKT; NF-κB: Nuclear factor kappa-light-chain-enhancer of activated B cells; JNK: c-Jun N-terminal kinase; PI3K: Phosphoinositide 3-kinase; GST: Glutathione-S-transferase; IAP: Inhibitor affinity purification; EGFR: Epidermal growth factor receptor; PI3KCA/B: Phosphoinositide 3-kinase catalytic subunit A/B; PI3KR1/2: Phosphoinositide 3-kinase regulatory subunit 1/2; ABL1: Abelson murine leukemia viral oncogene homolog 1; LFQ: Label free quantification; IC_50_: Half maximal inhibitory concentration; LC-MS/MS: Liquid chromatography tandem mass spectrometry.

## Competing interests

The authors declare that they have no competing interests.

## Authors’ contributions

The manuscript was written through contributions of all authors. All authors read and approved the final manuscript.

## Supplementary Material

Additional file 1 **Proteomic data.** List of captured SH2 proteins. Click here for file

Additional file 2 **Proteomic data.** Pull-down optimization. Click here for file

Additional file 3 **Detailed experimental procedures.** Experimental procedures and analytical data of the compound synthesis and supplementary figures and tables. Click here for file

Additional file 4 **Proteomic data.** Data set of the pervanadate depletion experiments. Click here for file

Additional file 5 **Proteomic data.** Data set of the dose response experiments. Click here for file
