# Supplementary material for: Probing SH2-domains using Inhibitor Affinity Purification (IAP)
Source: Proteome Sci. 2014 Jul 16;12:41. doi: 10.1186/1477-5956-12-41 (PMC4110944; doi:10.1186/1477-5956-12-41)
Supplement: Additional file 3 — Detailed experimental procedures. Experimental procedures and analytical data of the compound synthesis and supplementary figures and tables. [file 1477-5956-12-41-S3.pdf]

Supporting Information

Probing SH2 domains using inhibitor affinity purification (IAP)

Michael Höfener[1]*, Stephanie Heinzlmeir[2,3,4]*, Bernhard Kuster[2,5], Norbert Sewald[1§]

[1] Organic and Bioorganic Chemistry, Department of Chemistry, Bielefeld University, Universitätsstraße 25, 33615 Bielefeld, Germany

[2] Chair for Proteomics and Bioanalytics, Center of Life and Food Sciences Weihenstephan, Technische Universität München, Freising, Germany

[3] German Cancer Consortium (DKTK), Munich, Germany

[4] German Cancer Research Center (DKFZ), Heidelberg, Germany

[5] Center for Integrated Protein Science Munich, Freising, Germany

E-Mail: norbert.sewald@uni-bielefeld.de

Materials and methods for chemical synthesis

Chemicals and Solvents: Solvents were usually used as purchased in p.a. quality. Solvents of lower quality were purified by distillation. If necessary, methylene chloride was dried over calcium hydride. Toluene and tetrahydrofuran were dried over sodium. Acetonitrile for analytical and preparative HPLC was purchased in LiChrosolv quality from Merck. Water for this purpose was obtained from a Millipore MilliQ-Ultrapure water system. Chemicals were purchased at Acros, Sigma Aldrich, VWR and Alfa Aesar and were used without further treatment. Silica gel (0.04-0.063 mm) from Macherey-Nagel was used for flash chromatography.

NMR-spectroscopy: ^{1}H-NMR and ^{1}H,^{1}H-COSY experiments were recorded using a Bruker DRX 500 instrument (^{1}H-NMR: 500.1 MHz). ^{13}C-NMR, HSQC and HMBC experiments were recorded using a Bruker Avance 600 instrument (^{1}H-NMR: 600.1 MHz , ^{13}C-NMR: 150.9 MHz). The recorded data was processed using TopSpin v 2.1, the spectra were evaluated using MestReNova software 6.0.2. Deuterated chloroform was usually used as solvent for NMR measurements with tetramethylsilane (TMS) as internal standard. Other deuterated solvents were referenced to the solvent peak. Structures of unpublished molecules were assigned with the help of ^{1}H,^{1}H-COSY, HSQC and HMBC NMR experiments.

Mass-spectrometry: ESI-MS: ESI/APCI mass spectra were recorded using an Esquire 3000 ion trap mass spectrometer (Bruker Daltonik GmbH, Bremen, Germany) equipped with a standard ESI/APCI source. Samples were introduced by direct infusion with a syringe pump. Nitrogen served both as the nebulizer gas and the dry gas. Nitrogen was generated by a Bruker nitrogen generator NGM 11. Helium served as cooling gas for the ion trap and collision gas for MS^{n} experiments. The spectra were recorded with the Bruker Daltonik esquireNT 5.2 esquireControl software by the accumulation and averaging of several single spectra. DataAnalysis™ software 3.4 was used for processing the spectra.

MALDI-MS: MALDI experiments were performed using a Fourier Transform Ion Cyclotron Resonance (FT-ICR) mass spectrometer APEX III (Bruker Daltonik GmbH, Bremen, Germany) equipped with a 7.0 T, 160 mm bore superconducting magnet (Bruker Analytik GmbH – Magnetics, Karlsruhe, Germany), infinity cell, and interfaced to an external MALDI ion source. Nitrogen served both as the nebulizer gas and the dry gas for ESI. Nitrogen was generated by a Bruker nitrogen generator NGM 11. Argon served as cooling gas in the infinity cell and collision gas for MS_n experiments. Scan accumulation and fourier transformation were performed with XMASS NT (7.08) on a PC Workstation, for further data processing DataAnalysis™ 3.4 was used.

LC-MS: LC-MS analysis was accomplished using a Waters Alliance HT equipped with a Waters Symmetry 3.5 µm column (C_8, 100 x 2.1 mm, eluent A H_2O/HCOOH = 100:0.1, eluent B CH_3CN/HCOOH = 100:0.1, flowrate 0.4 mL·min-1 using a gradient from 5-95% B over 10 minutes) coupled with a Waters micromass ZQ2000 ESI-MS.

Analytical Reversed Phase-High Performance Liquid Chromatography (RP-HPLC)

Thermo Separation Products System: Surveyor Autosampler Plus, Surveyor LC Pump Plus, Surveyor PDA Plus Detector (190 -800 nm, continous); column: ET 125/4 Nucleosil 100-5 C_{18} PPN; software: ChromQuest 5.0; eluent A: 5 % H_2O, 95 % ACN, 0.05 % TFA; eluent B: 95 % H_2O, 5 % ACN, 0.05 % TFA.

Supplementary table 1: Analytical HPLC method, λ = 192-480 nm.

time [min]	eluent A	eluent B	flow [mL min^{-1}]
**1**	0	100	0.75
**2**	0	100	0.75
**4**	100	0	0.75
**4**	0	100	0.75

Preparative Reversed Phase - High Performance Liquid Chromatography (RP-HPLC)

Hitachi MERCK LaChrom system: LC 7150 pump; UV-Vis L 7420 detector; precolumn: Vydac high-performance guard column (C_{18}); column: Phenomenex Jupiter 10 µ 300 Å (C_{18}; 250 x 21.2 mm); eluent A: 5 % H_2O, 95 % ACN, 0.05 % TFA; eluent B: 95 % H_2O, 5 % ACN, 0.05 % TFA.

Supplementary table 2: Preparative HPLC method, λ = 254 nm.

time [min]	eluent A	eluent B	flow [mL min^{-1}]
**0**	0	100	10
**5**	0	100	10
**40**	100	0	10
**50**	0	100	10

Synthesis of the F2pmp-probe 1

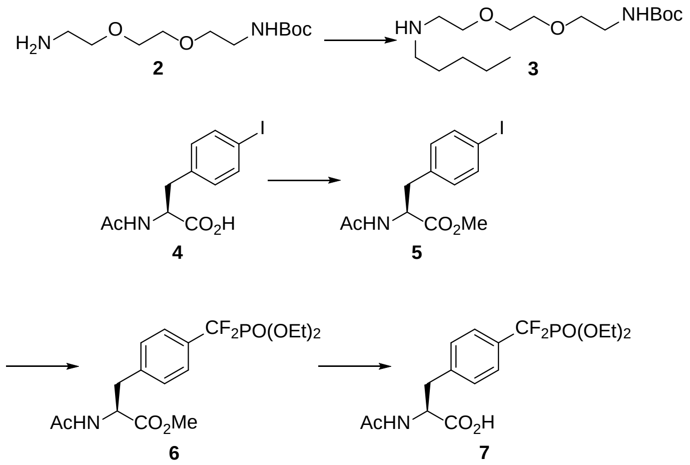

Supplementary Scheme 1: Overview of the synthesis pathway for the F2pmp building block **7**.

***tert*-Butyl 2-(2-(2-aminoethoxy)ethoxy)ethylcarbamate (2)** (J. Chem. Soc., Dalton Trans., 2000, 1805-1812) A mixture of Boc_2O (2.9 g, 14 mmol) in THF (30 mL) was added dropwise to a solution of 2-(2-(2-aminoethoxy)ethoxy)ethanamine (14.5 g, 98 mmol) in THF (30 mL) over a period of 5 h. The resulting suspension was stirred overnight at rt prior evaporation of the solvent. The residue was dissolved in water (50 mL) and extracted with DCM (3x20 mL). The organic phases were washed with water (10 mL) dried over $MgSO_4$ and the solvent was evaporated. The crude product was used without further purification in the next step. **Formula:** $C_{11}H_{24}N_2O_4$ (M = 248.3). **Yield:** 3.3 g (13.3 mmol, 95%). **^{1}H-NMR** (500 MHz, $CDCl_3$): δ [ppm] = 5.16 (s, 1H, N**H**), 3.62 (s, 4H, 2xC**H**$_2$), 3.54 (m, 4H, 2xC**H**$_2$), 3.32 (m, 2H, C**H**$_2$), 2.90 (t, 2H, *J* = 5.1 Hz, C**H**$_2$), 1.44 (s, 9H, 3xC**H**$_3$).

***tert*-Butyl 2-(2-(2-(pentylamino)ethoxy)ethoxy)ethylcarbamate (3)** Amine **2** (2.5 g, 10 mmol) and DIPEA (1.75 mL, 10 mmol) were prearranged in dry DCM (6 mL). A solution of 1-bromopentane (623 µL, 5 mmol) in DCM (1.5 mL) was added at rt over 4 h. After stirring at rt for 12 h the reaction mixture was dissolved in 1M NaOH solution and extracted with DCM (3x10 mL). The combined organic layers were dried over $MgSO_4$ and the solvent evaporated to give the crude product that was purified using flash chromatography (eluent: DCM/MeOH 95:5 +1% TEA) to give the title compound **3**. **Formula:** $C_{16}H_{34}N_2O_4$ (M = 318.4). **Yield:** 620 mg (1.94 mmol, 39%). **^{1}H-NMR** (500 MHz, CDCl3): δ [ppm] = 5.15 (s, 1H, N**H**), 3.74-2.79 (m, 12H, C**H**$_2$), 1.65 (s, 1H, N**H**), 1.43 (s, 9H, C(C**H**3)3), 1.38-1.31 (m, 8H, 4xC**H**2), 0.89 (t, 3H, C**H**3). **^{13}C-NMR** (125 MHz, CDCl3): δ [ppm] = 156.3, 79.4, 70.5, 68.6, 49.3, 46.1, 40.5, 29.3, 28.6, 28.1, 22.5, 14.1, 9.2.

(S)-2-Acetamido-3-(4-iodophenyl)propanoic acid (4) 4-Iodophenylalanine (764 mg, 2.6 mmol) was dissolved in Ac_2O (30 mL) and treated with TEA (368 µL, 266 mg, 2.6 mmol). The reaction mixture was stirred at rt for 12 h prior to hydrolysis of the excess anhydride with water. The resulting suspension was concentrated to a volume of 5 mL and dissolved in 5% $KHSO_4$ solution (40 mL). The aqueous phase was extracted with EE (4x10 mL) and the combined organic phases were dried over $MgSO_4$. The solvent was evaporated and the crude product was used in the next step without further purification. **Formula:** $C_{11}H_{12}INO_3$ (M = 333.1). **Yield:** 925 mg (2.8 mmol, quant.), **^{1}H-NMR** (500 MHz, $(CD_3)_2SO$): δ [ppm] = 12.67 (s, 1H, CO_2**H**), 8.16 (d, 1H, *J* = 8.3 Hz, N**H**), 7.62 (d, 2H, *J* = 8.6 Hz, Ar-**H**), 7.03 (d, 2H, *J* = 8.7 Hz, Ar-**H**), 4.38 (ddd, 1H, *J* = 4.9, 8.4, 9.3 Hz, C_α-**H**), 2.99 (dd, 1H, *J* = 5.0, 14.0 Hz, C_β-

H), 2.79 (dd, 1H, *J* = 9.4, 14.0 Hz, $C_β$-**H**), 1.77 (s, 3H, C**H**3). **^{13}C-NMR** (125 MHz, $(CD_3)_2SO$): δ [ppm] = 172.8 (**C**O_2H), 169.0 (**C**ON), 137.4 (Ar**C**), 136.6 (2xAr**C**), 131.3 (2xAr**C**), 92.1 (Ar**C**), 53.1 (**C**$_α$), 36.2 (**C**$_β$), 20.3 (**C**H_3). **ESI-MS:** $m\cdot z^{-1}$ = 334.0 $[M+H]^+$(calc. 333.99).

(S)-Methyl 2-acetamido-3-(4-iodophenyl)propanoate (5) (J. Med. Chem. 2007, 50, 856-864) Carboxylic acid **4** (925 mg, 2.8 mmol) was dissolved in MeOH (10 mL) and $SOCl_2$ (404 µL, 661 mg, 5.6 mmol) was added dropwise over 5 min at 4 °C. After stirring at rt for 2 h H_2O (10 mL) was added carefully and the reaction mixture neutralized with 1M NaOH. MeOH was evaporated and the residue extracted with EE (5x15 mL). The combined organic layers were washed with 5% $KHSO_4$ solution, sat. $NaHCO_3$ solution and brine prior to drying over $MgSO_4$ and evaporation of the solvent. The title compound **5** was used as obtained in the next step. **Formula:** $C_{12}H_{14}INO_3$ (M = 347.2). **Yield:** 634 mg (1.8 mmol, 67%), **ESI-MS:** $m\cdot z^{-1}$ = 348.1 $[M+H]^+$(calc. 348.0).

(S)-Methyl 2-acetamido-3-(4-((diethoxyphosphoryl)difluoromethyl)phenyl)propanoate (6) (Tetrahedron 1997, 53, 3, 815-822) HCl activated Zn (279 mg, 4.3 mmol) was dissolved in DMAC (1 mL) and treated with diethyl(bromodifluoromethyl)phosphonate (760 µL, 1.1 g, 4.3 mmol). The exothermic reaction was stirred for 2 h without cooling prior to addition of CuBr (614 mg, 4.3 mmol) in one portion. The reaction mixture was stirred at rt for 30 min followed by addition of aryl iodide **5** (634 mg, 1.8 mmol). After stirring at rt for 3 d the reaction mixture was dissolved in EE (20 mL) and washed with 5% $KHSO_4$ solution (2x5 mL), sat. $NaHCO_3$ solution (2x5 mL) and brine (1x5 mL). The organic phase was dried over $MgSO_4$ and the solvent evaporated to give the crude product that was purified using flash chromatography (eluent: DCM/MeOH 99:1) to give the title compound **6**. Unreacted starting material was recovered. **Formula:** $C_{17}H_{24}F_2NO_6P$ (M = 407.4). **Yield:** 102 mg (0.25 mmol, 14%), **^{1}H-NMR** (500 MHz, $CDCl_3$): δ [ppm] = 7.55 (d, 2H, *J* = 7.7 Hz, Ar-**H**), 7.19 (d, 2H, *J* = 8.3 Hz, Ar-**H**), 5.92 (d, 1H, *J* = 7.8 Hz, N**H**) 4.91 (ddd, 1H, *J* = 5.8, 5.8, 7.6 Hz, $C_α$-**H**), 4.08-4.26 (m, 4H, 2xC**H**$_2$), 3.21 (dd, 1H, *J* = 6.4, 14.0 Hz, $C_β$-**H**), 3.13 (dd, 1H, *J* = 5.7, 14.0 Hz, $C_β$-**H**), 3.72 (s, 3H, C**H**$_3$), 1.91 (s, 3H, C**H**$_3$) 1.31 (t, 3H, *J* = 6.9Hz, C**H**$_3$), 1.30 (t, 3H, *J* = 6.9Hz, C**H**$_3$). **ESI-MS:** $m\cdot z^{-1}$ = 408.1 $[M+H]^+$(calc. 408.1).

(S)-2-Acetamido-3-(4-((diethoxyphosphoryl)difluoromethyl)phenyl)propanoic acid (7) (Tetrahedron 1997, 53, 32, 11171-11178) Methyl ester **6** (102 mg, 0.25 mmol) was dissolved in a mixture of MeOH/THF/H_2O (1:1:1, 2 mL) and treated with LiOH (12.6 mg, 0.3 mmol). The reaction mixture was stirred at rt for 30 min and subsequently quenched with formic acid (1 mL). Volatile components were evaporated and the residue purified using preparative RP-HPLC to give the title compound **7**. **Formula:** $C_{16}H_{22}F_2NO_6P$ (M = 393.3). **Yield:** 77 mg (0.196 mmol, 78%). **^{1}H-NMR** (500 MHz, $CDCl_3$): δ [ppm] = 8.04 (s, 1H, CO_2**H**), 7.51 (d, 2H, *J* = 8.2 Hz, Ar-**H**), 7.22 (d, 2H, *J* = 8.4 Hz, Ar-**H**), 6.68 (d, 1H, *J* = 7.4 Hz, N**H**), 4.91 (ddd, 1H, *J* = 5.8, 5.8, 7.7 Hz, $C_α$-**H**), 4.06-4.31 (m, 4H, 2xC**H**$_2$), 3.24 (dd, 1H, *J* = 5.0, 14.0 Hz, $C_β$-**H**), 3.19 (dd, 1H, *J* = 6.0, 14.0 Hz, $C_β$-**H**), 1.92 (s, 3H, C**H**$_3$), 1.34 (t, 3H, *J* = 6.9Hz, C**H**$_3$), 1.31. (t, 3H, *J* = 6.9Hz, C**H**$_3$). **^{13}C-NMR** (125 MHz, $CDCl_3$): δ [ppm] = 172.4 (**C**O_2H), 170.5 (**C**ON), 138.9 (Ar**C**), 129.0 (4xAr**C**), 125.9 (Ar**C**-CF_2), 64.8 (**C**H_2-CH_3), 52.2 (**C**$_α$), 36.4 (**C**$_β$), 22.1 (**C**H_3), 15.7 (CH_2-**C**H_3).

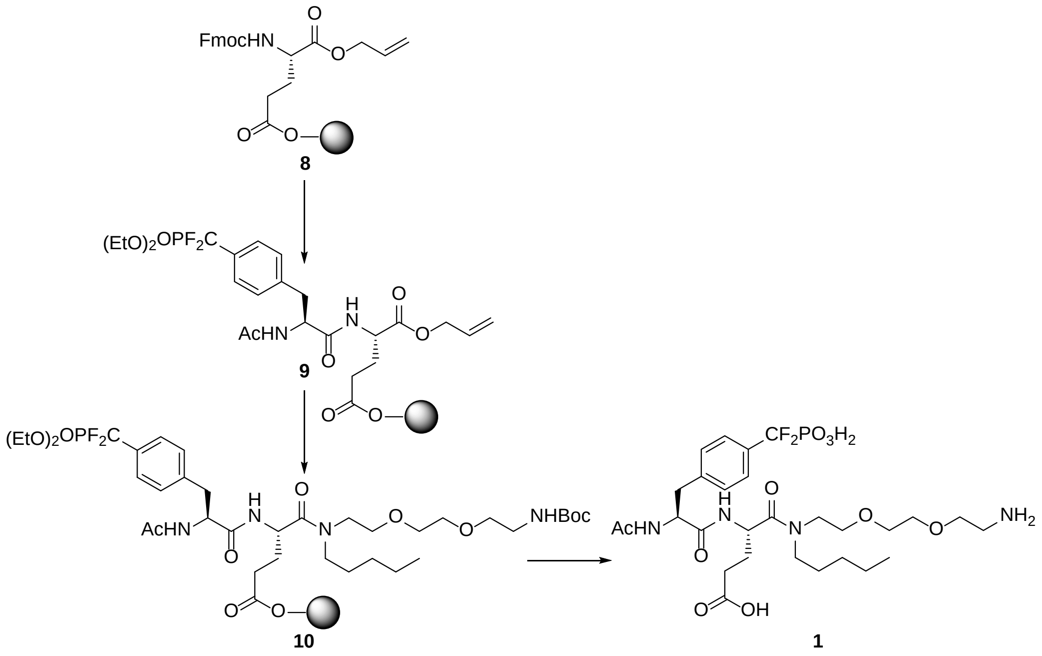

Supplementary Scheme 2: Overview of the synthesis pathway for the F2pmp probe **1**.

(S)-4-((S)-2-Acetamido-3-(4-(difluoro(phosphono)methyl)phenyl)propanamido)-5-((2-(2-(2-aminoethoxy)ethoxy)ethyl)(pentyl)amino)-5-oxopentanoic acid (1) (adopted according to Vu et al, Current Medicinal Chemistry, 2000, 7, 1081-1100) The F2PmP probe **1** was synthesized using standard SPPS procedures on 2-chlorotrityl resin. The resin (500 mg) was incubated with a solution of Fmoc-L-Glu-OAll (614 mg, 1.5 mmol) and DIPEA (523 µL, 3 mmol) in DCM (5.0 mL) for 2 h at rt under argon atmosphere. The coupling solution was removed by filtration and the resin washed with DMF (5x10 mL) and DCM (5x10 mL). After incubation with MeOH (10 mL) for 10 min at rt the solvent was removed by filtration and the resin dried under reduced pressure. The glutamate loaded 2-chlorotrityl resin **8** (0.973 mmol/g, 135 mg, 0.131 mmol) was treated with piperidine solution (20% in DMF, 3x30 min) and subsequently washed according to the standard washing procedure. For coupling the F_2pmp-building block **7** the resin was incubated with carboxylic acid **7** (77 mg, 0.196 mmol), HATU (75 mg, 0.196 mmol), HOAt (27 mg, 0.196 mmol) and DIPEA (50 mg, 65 µL, 0.392 mmol) in DMF (2 mL) for 12 h at rt. The resin **9** was washed using the standard washing procedure and subsequently incubated with a solution of phenylsilane (485 µL, 425 mg, 3.900 mmol) and $Pd(PPh_3)_4$ (31 mg, 0.026 mmol) in DMF (2 mL) 2x60 min at rt. After washing analogous to the previous steps the resin was incubated with a solution of secondary amine **3** (125 mg, 0.392 mmol), HATU (150 mg, 0.392 mmol), HOAt (54 mg, 0.392 mmol) and DIPEA (100 mg, 130 µL, 0.784 mmol) in DMF (2 mL) for 12 h at rt. After standard washing the peptide **10** was cleaved using a solution of TFA/TIS/H_2O (95:2.5:2.5, 4 mL) 5x30 min. After washing with ACN (4 mL) the cleaving fractions and the ACN fraction were combined and the volatile components removed via freeze drying. The residue was dissolved in a solution of 50% (v/v) TMSBr in DCM and stirred at rt until complete cleavage of the ethyl groups. Volatile components were removed under reduced pressure and the residue purified using preparative RP-HPLC to give the title compound **1**. **Formula:** $C_{28}H_{45}F_2N_4O_{10}P$ (M = 666.7). **Yield:** 12 mg (0.02 mmol, 15%), **^{1}H-NMR** (600 MHz, H_2O/D_2O, 50 °C, water suppression): δ [ppm] = 8.30-8.48 (m, 1H), 8.07-8.28 (m, 1H), 7.28-7.91 (m, 2H), 7.58-7.72 (m, 2H), 3.88-4.11 (m, 10H), 3.61-3.81 (m, 2H), 3.47-3.58 (m, 2H), 3.31-3.47 (2, 2H), 2.63-2.75 (m, 1H), 2.44-2.54 (m, 1H), 2.35-2.44 (m, 2H), 2.25-2.35 (m, 3H), 2.08-2.24 (m, 1H), 1.97-2.08 (m, 1H), 1.88-1.97 (m, 1H), 1.75-1.87 (m, 1H), 1.52-

1.69 (m, 4H), 1.45-1.52 (m, 1H), 1.13-1.25 (m, 2H). The NMR data consists of 8 sets of signals, likely due to rotation of all amide bonds that show chemical exchange in ROESY-NMR. **Exact mass (ESI):** mz^{-1} = 689.27147 $[M+Na^{+}]^{+}$, calculated: 689.27336.

Data from the NCI-60 cell line panel

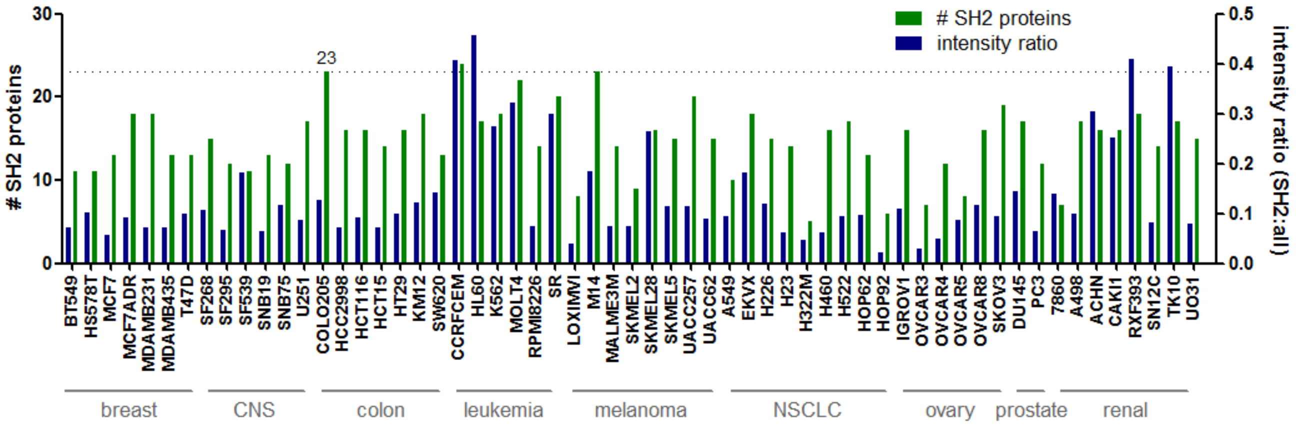

Supplementary Figure1: Data from a proteome analysis of the NCI-60 cell line panel. The number of SH2 proteins (left axis, green) and the intensity ratio of SH2 proteins (right axis, blue). The number of SH2 proteins was considered to be a more important criteria than the intensity ratio.

Basis for Figure 3 in the manuscript

Supplementary Table3: Comparison to full proteome data of cell lines OVCAR8, K562, SKNBE2 and COLO205. The shown values correspond to the summed intensities of the MS experiment, ratio based on raw summed intensities. The highest SH2 protein ratio (SH2 probe enrichment, 10% Glycerol) of 7.8% is highlighted in red.

			# proteins		Log10 (Intensity)		ratio
	**Cell line**	**pull-down optimization**	**# all proteins**	**# SH2 proteins**	**all proteins**	**SH2 proteins**	**Ratio in %**
**Full proteome**	**Colo205**	**-**	**5444**	**32**	**12.73**	**9.83**	**0.13**
	**K652**	**-**	**4716**	**23**	**12.01**	**9.26**	**0.18**
	**Ovcar8**	**-**	**5191**	**23**	**12.53**	**9.34**	**0.06**
	**SKNBE2**	**-**	**5222**	**22**	**12.6**	**9.53**	**0.09**
**SH2 probe enrichment**	**Cell mix**	**-**	**1557**	**22**	**10.23**	**8.66**	**2.70**
	**Cell mix**	**0.1% SDS**	**1092**	**13**	**9.43**	**7.80**	**2.30**
	**Cell mix**	**10% Glycerol**	**1168**	**20**	**9.60**	**8.49**	**7.80**
	**Cell mix**	**650 nM NaCl**	**1196**	**16**	**9.76**	**7.88**	**1.31**
**Blocked beads**	**Cell mix**	**-**	**1152**	**11**	**9.42**	**6.27**	**0.07**

Distribution of SH2 proteins

	full proteome					SH2 enrichment			
	COLO 205	K562	OVCAR8	SKNBE2	blocked	normal	0.1% SDS	650nM NaCl	10% glycerol
GRB2	1	2	2	5	2	12	7	6	11
CRKL	7	1	5	3	3	1	8	5	1
CSK	5	8	9	9	6	6	1	11	12
LYN	1	1	19	1	5	2	13		2
ABL1		11		22	1	13	4	15	8
TNS4	4				8	2	2	1	1
PTPN6	19				1	8	5	4	13
PIK3R1	3		18			1	1	2	2
SRC	13		13		7		11	8	14
PIK3R2	32		22			3	3	3	3
BTK		4			9	9	6	9	
STAT3	6	6	17	4		17			
YES1		14	7		4	11			19
FYN	23		21	19		16			16
STAT1	2	5	3	2					
PTPN11	8	7	4	6					
CRK	12	9	1	14					
TNS3	24					7		12	5
SYK	2					5		1	17
RASA1	11	22	12	13					
GRB7	16		23					16	4
PLCG1	25	16	11	7					
SHCBP1	27	17	8	11					
INPPL1	26	13	1	17					
SHC1	17	19	2	12					
TNS1							9	7	6
VAV2	9		6	8					
LCK	3				11	15			
PIK3R3							12	13	15
PTK6						21		14	7
TEC		12				18			18
NCK1	22		14	15					
NCK2	29		16	18					
STAT2	31	2		2					
STAT5A		3				19			
FRK	14					22			
BCAR3		23	15						
FES						4			
FER									9
CHN1				1					
HCK						14			
INPP5D	15								
STAT5B		15							
SHD				16					
STAT6	18								
GRB1		18							
SH2D4A	21								
SOCS2		21							
ABL2				21					
SHB	28								

Supplementary Figure 2: Distribution of SH2 proteins in the used cell lysates. It is not possible to give comparable quantitative values in this list, since full proteome and pull-downs were quantified with different biochemical methods. Therefore, the “abundance” is depicted as heatmap illustrating the rank of the protein within the respective sample.

IC_{50}-values from the competition experiment

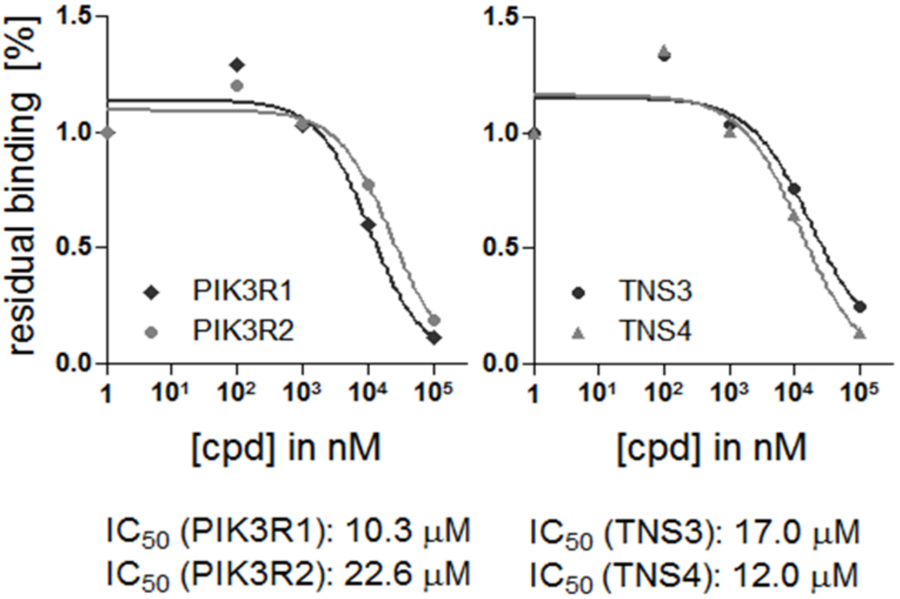

Supplementary Figure 3: IC_{50}-values of the PI3K subunits PI3KR1 and PI3KR2 and the tensins TNS3 and TNS4.

Statistical analysis of the pervanadate depletion experiments

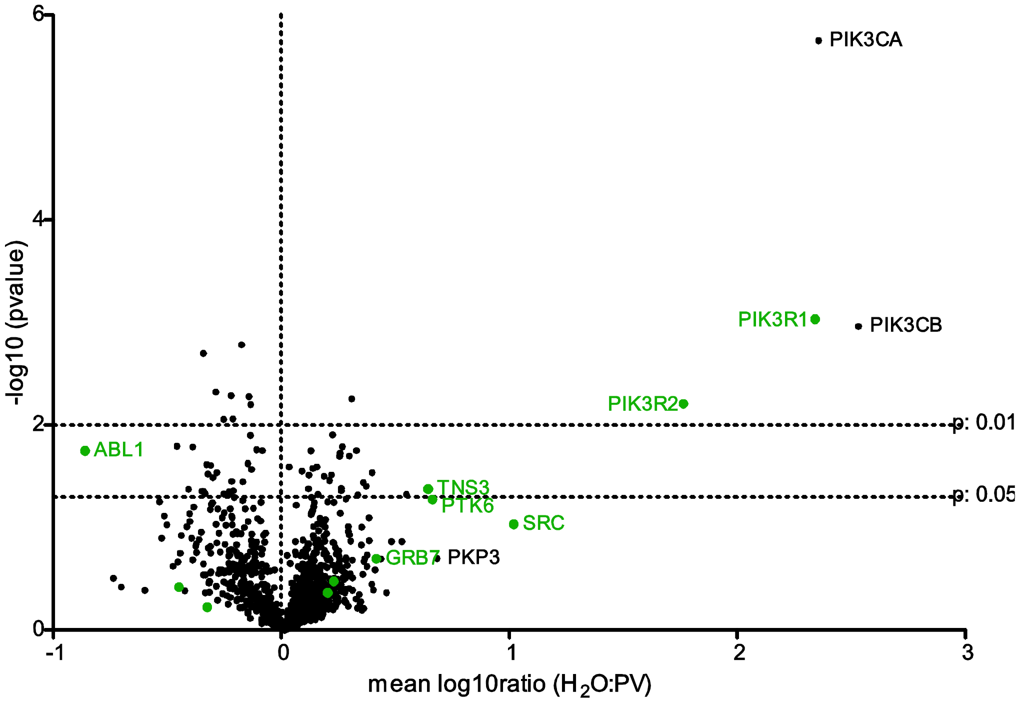

Supplementary Figure 4: Volcano plot of the pull-down data from the pervanadate depletion experiments.
